# Case Report: Recurrent stroke-like episodes triggered by high-altitude exposure in X-linked charcot-marie-tooth disease

**DOI:** 10.3389/fgene.2026.1769172

**Published:** 2026-02-12

**Authors:** Jing Zhong, Tang Yang, Bo Wu, Shuai Jiang

**Affiliations:** Department of Neurology, West China Hospital, Sichuan University, Chengdu, Sichuan, China

**Keywords:** Charcot–Marie–Tooth disease (CMTX), GJB1 gene, high-altitude hypoxia, reversible white matter lesions, stroke-like episodes

## Abstract

X-linked Charcot-Marie-Tooth disease type 1 (CMTX1) is a rare inherited neuropathy caused by mutations in the GJB1 gene, leading to progressive distal muscle weakness and atrophy. In this case study, a 37-year-old man presented with recurrent episodes of numbness involving the lips, right hand, and foot, followed by right-sided limb weakness and dysarthria four times over the past 20 years. Notably, the last three episodes were consistently triggered within 3 days after descent to sea level following exposure to high-altitude environments (8,500–10,000 feet, with transit above 13,000 feet). Based on the neurologic examination, brain magnetic resonance imaging (MRI), and genetic testing, the patient was diagnosed with X-linked Charcot-Marie-Tooth disease. This case underscores the importance of considering high-altitude exposure as a potential trigger and highlights the value of GJB1 testing in young patients presenting with acute stroke-like episodes and signs of peripheral neuropathy.

## 1 Introduction

X-Linked Charcot-Marie-Tooth Disease (CMTX) is an X-linked hereditary disease characterized by a progressive sensorimotor neuropathy. X-linked Charcot-Marie-Tooth disease type 1 (CMTX1) is the most common form of CMTX, mainly caused by mutations in the gap junction protein beta-1 gene (GJB1), which encodes the protein connexin-32 (Cx32) (Saporta et al., 2011; Lu et al., 2017). Cx32 is widely expressed in in Schwann cells of the peripheral nervous system and in myelinating oligodendrocytes within the central nervous system (CNS) (Rash et al., 2001). Clinically, CMTX1 typically presents in childhood or adolescence with slowly progressive distal muscle weakness, atrophy, foot deformities, reduced or absent deep tendon reflexes, and sensory deficits. These features reflect chronic sensorimotor neuropathy of peripheral nerves (Fridman et al., 2015). In recent years, an increasingly recognized feature of CMTX1 is its association with transient, stroke-like CNS episodes (Wang and Yin, 2016). These acute neurological events, characterized by focal weakness, dysarthria, and numbness, can be challenging to distinguish from acute cerebrovascular disease, often leading to initial misdiagnosis (Zhong et al., 2012; Nicholson and Pulst, 2017). Herein, we present a case of CMTX1 with recurrent stroke-like episodes, providing a clinical example of this diagnostic challenge.

## 2 Case presentation

A 37-year-old Chinese male presented with a 20-year history of recurrent neurological symptoms, including numbness of the lips, right hand, and right foot, followed by right-sided limb weakness and dysarthria. The first episode occurred at age 16, when he was diagnosed with “acute stroke” at a local hospital; symptoms fully resolved within 2 weeks with unspecified symptomatic treatment. He remained asymptomatic until October 2022, when he experienced three recurrent episodes within 3 days of returning to sea level from high-altitude regions (8,500–10,000 feet, with transit above 13,000 feet). Each episode lasted 1–5 h and mirrored the initial presentation, resolving without residual deficits.

**FIGURE 1:**
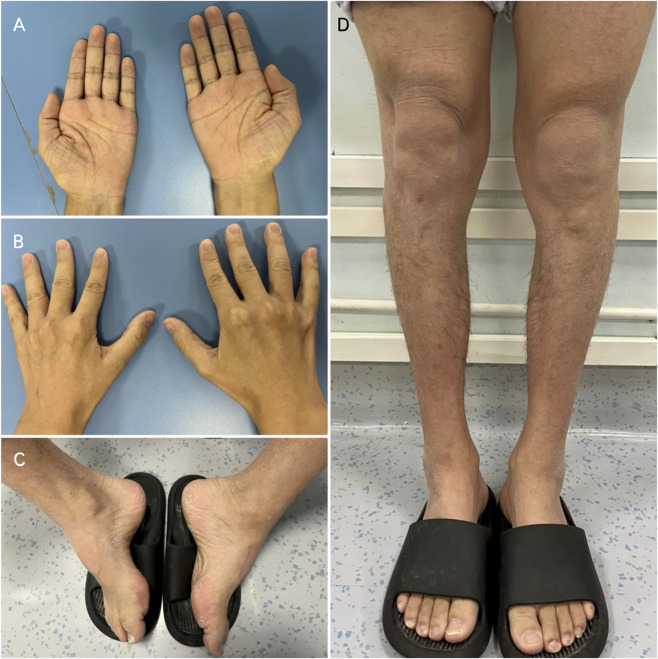
The patient’s hands and lower limbs. **(A,B)** Atrophy of the thenar, hypothenar, and interosseous muscles. **(C)** Pes cavus. **(D)** Muscle atrophy in the distal portion of the lower limbs.

**FIGURE 2:**
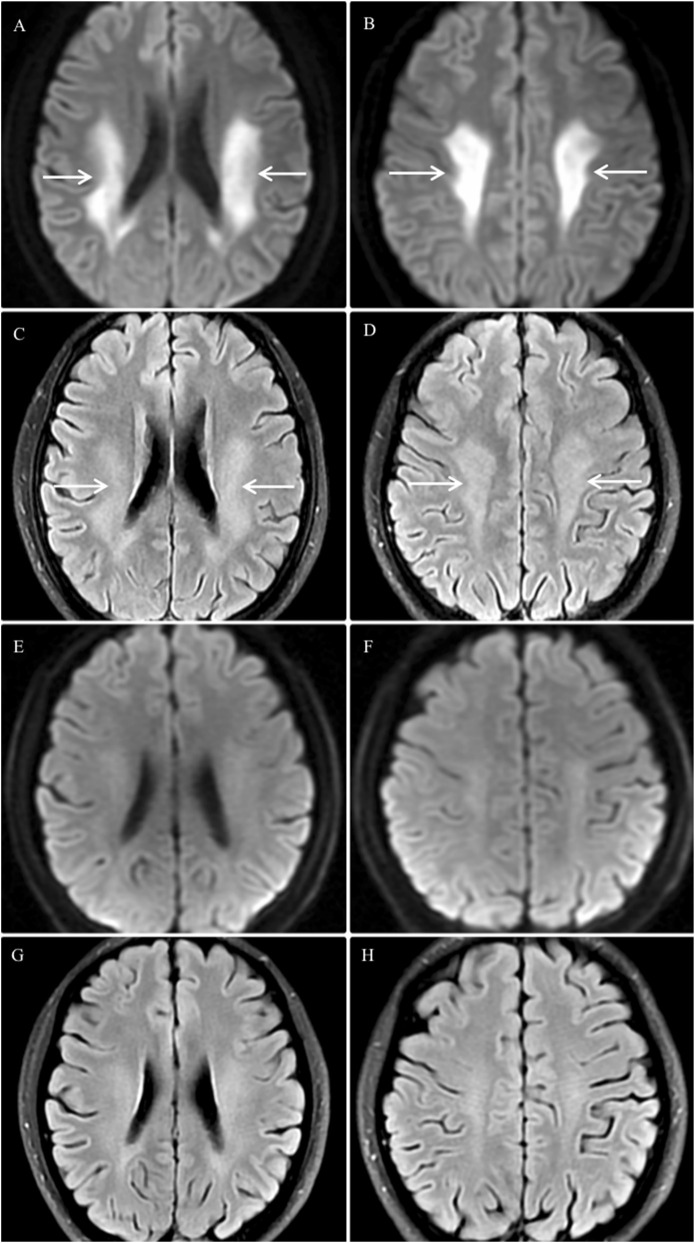
Brain MRI findings. **(A–D)** Acute Phase: Brain MRI on the third day after the final episode demonstrated bilateral symmetric restricted diffusion (arrows) in the periventricular deep white matter and centrum semiovale on DWI **(A,B)**, with corresponding hyperintensity (arrows) on FLAIR images **(C,D)**. **(E–H)** Resolution Phase: DWI **(E,F)** and FLAIR **(G,H)** sequences obtained 1 month later showed near-complete resolution of the aforementioned abnormalities.

Neurological examination showed absent or decreased deep tendon reflexes in all four limbs, muscle atrophy in the hands and distal portion of the lower limbs, and pes cavus (Figure 1). Needle Electromyography (EMG) and Nerve Conduction Studies (NCS) performed after the last episode revealed chronic neurogenic damage in the right tibialis anterior, left vastus medialis, and right first dorsal interosseous muscles. The overall electrophysiological profile indicates significant peripheral nerve impairment affecting both the upper and lower limbs, with involvement of sensory and motor fibers. The pattern is predominantly demyelinating with associated axonal damage. No stenosis of cerebral arteries was detected on Digital Subtraction Angiography (DSA). Brain magnetic resonance imaging (MRI) performed on day 3 after the most recent episode revealed bilateral symmetric restricted diffusion in the periventricular deep white matter, centrum semiovale, and the splenium of the corpus callosum on diffusion-weighted imaging (DWI) sequence, with corresponding hyperintensity on fluid-attenuated inversion recovery sequence (FLAIR). A follow-up Brain MRI 1 month later showed near-complete resolution of these abnormalities (Figure 2). Notably, his mother had pes cavus, but no other family members reported neuropathy or recurrent neurological symptoms. Given the clinical manifestations and imaging findings, the patient was referred to medical genetics due to suspected X-Linked Charcot-Marie-Tooth Disease (CMTX). Genetic testing revealed a novel-frameshift mutation in exon 2 of the GJB1gene: c.256_257insCCCCATCTCCCATGTGCGGCTGTGGTCCCTGCAGCTCATCCTAGTTTCCA (p.Ala88fsTer13). This variant, which has not been catalogued in major genomic databases (dbSNP, gnomAD, HGMD, ClinVar) or described in the literature, represents a novel mutation that confirms the diagnosis of CMTX1.

## 3 Discussion

### 3.1 High-altitude-triggered CNS episodes and mechanism insights

In this case report, we discussed the experience of a 37-year-old man who was diagnosed with X-linked Charcot-Marie-Tooth disease type 1(CMTX1) after presenting with recurrent, transient stroke-like episodes triggered by high-altitude exposure. The transient CNS symptoms in CMTX1 are frequently precipitated by metabolic stressors, including high-altitude exposure, fever, or hyperventilation. In a systematic review of 47 CMTX1 cases, 63.8% had identifiable triggers: fever/infection (27.7%), high-altitude exposure (12.8%), and intense exercise (8.5%) (Tian et al., 2020).

To further characterize this specific presentation, we aggregated clinical, radiological, and genetic data from seven male CMTX1 patients (including the present case) whose symptoms were triggered by high altitude (Table 1). (Paulson et al., 2002; Al-Mateen et al., 2014; Hanemann et al., 2003; Hardy et al., 2019; Sagnelli et al., 2014) The age of these patients ranged from 10 to 37 years. The high altitude was above 4,000 feet in four patients (57%), approximately 2,500 feet in one patient, and unspecified in the remaining two patients. Clinical presentations included right hemiparesis (57%), tetraparesis (29%), and symmetric weakness (14%). All neurological deficits resolved completely or nearly within 3 h to 2 weeks. A consistent radiological feature was the presence of bilateral T2/FLAIR hyperintensities in the cerebral white matter, most commonly involving the splenium of the corpus callosum (57%) and the centrum semiovale (57%). In the four cases where DWI was reported (not shown), these areas showed corresponding restricted diffusion. Follow-up MRIs obtained from day 6–18 months later showed marked improvement or normalization of these lesions. Genetically, five cases had missense GJB1 mutations and two had frameshift mutations (no clear genotype–phenotype distinction in clinical course).

**TABLE 1:** Published and current cases of CMTX1 with high-altitude–provoked CNS episodes.

References	Demographics	Maximal altitude/Feet	Motor impairment	Duration to recovery	T2/FLAIR hyperintensity site	Follow-up result	Follow-up interval	Nucleotide variation
Paulson et al. (2002)	16/male	8,000	tetraparesis	10 h	pericallosal	Normal	2 m	c.565G>A
Paulson et al. (2002)	26/male	11,000	symmetrical weakness	2 w	posterior, corpus callosum splenium	Normal	1 y	-
Al-Mateen et al. (2014)	14/male	4,025	right hemiparesis	3 d	centrum semiovale	Improved	2 m	c.260C>G
Hanemann et al. (2003)	10/male	absent *	tetraparesis; hemiparalysis	3 h	centrum semiovale; cerebellar peduncle	Improved	3 m	c.304_306delGAG
Hardy et al. (2019)	13/male	absent *	right hemiparesis	2 d	centrum semiovale, posterior corona radiata, corpus callosum splenium	-	-	c.227T>C
Sagnelli et al. (2014)	16/male	2,500	right hemiparesis	2 d	corpus callosum splenium, corticespinal tracts, cerebellar peduncle	Normal	18 m	c.297_298insCAA
This case	37/male	10,000	right hemiparesis	3 d	periventricular, centrum semiovale, corpus callosum splenium	Normal	1 m	c.256_257ins [52bp]

* The article did not specify the exact altitude or location, only mentioning that the patient had been to a mountainous area prior to the onset of symptoms.

These reversible CNS episodes occurred independently of the chronic peripheral neuropathy typically associated with CMTX1, suggesting a distinct pathophysiological mechanism. A proposed mechanism for high-altitude-induced CNS symptoms in CMTX1 involves transient cerebrospinal fluid (CSF) acidification secondary to hypoxia (Paulson et al., 2002). High-altitude hypoxia may trigger hyperventilation, leading to respiratory alkalosis. As a compensatory response, CSF bicarbonate concentration is thought to fall. Upon return to sea level, ventilation normalizes rapidly, causing a swift rise in arterial carbon dioxide, while CSF bicarbonate recovers more slowly, likely producing a transient CSF acidification. This acidotic shift inhibits pH-sensitive CNS gap junctions, causing their temporary closure (Abrams et al., 2003; Oshima, 2014). Normally, these alternative gap junctions facilitate the exchange of ions and small metabolites, partially compensating for dysfunctional Cx32 gap junctions caused by GJB1 mutations (Wasseff and Scherer, 2011). This compensatory capacity likely explains the general absence of CNS symptoms in baseline conditions. Nevertheless, the acidotic closure of these gap junctions may acutely impairs communication between astrocytes and oligodendrocytes, disrupting fluid and ion homeostasis. Clinically, this manifests as episodic limb weakness and dysarthria, while radiologically, it presents as reversible restricted diffusion on DWI. It is hypothesized that other metabolic stressors, such as hyperventilation or intense exercise, may trigger symptoms through a similar acidification-mediated mechanism. The self-limiting nature of these episodes underscores a dynamic, non-destructive pathophysiology, in which both symptoms and imaging abnormalities typically resolve once the metabolic stressor is removed and cellular homeostasis is restore.

### 3.2 Neuroimaging features

A hallmark of CMTX1-related CNS involvement is the presence of bilateral, symmetric white matter lesions on MRI, typically localized to the periventricular region, centrum semiovale, or splenium of the corpus callosum. These lesions often demonstrate restricted diffusion in the acute phase and resolve nearly completely on follow-up imaging (Al-Mateen et al., 2014; Wang and Yin, 2016; Tian et al., 2020). When such a distinctive radiologic pattern is identified—particularly in patients with clinical features of peripheral neuropathy such as pes cavus or reduced reflexes—a detailed family history should be obtained to assess for a hereditary neuropathy. In these scenarios, CMTX1 should be strongly suspected, and genetic testing for GJB1 mutations is recommended.

### 3.3 Differential diagnosis of transient CNS episodes

The differential diagnosis for these transient CNS episodes includes transient ischemic attack (TIA), mitochondrial encephalopathy with lactic acidosis and stroke-like episodes (MELAS), acute disseminated encephalomyelitis (ADEM), and periodic paralysis (Basu et al., 2011; Nicholson and Pulst, 2017; Tian et al., 2020). Several key features help distinguish CMTX1 from these conditions. Unlike TIA, CMTX1-related lesions are typically symmetric and reversible, and do not conform to vascular territory distribution (Fitzpatrick et al., 2019). MELAS often exhibits stroke-like lesions that cross vascular boundaries, but is distinguished by elevated serum lactate, progressive clinical course (El-Hattab et al., 2015). ADEM usually follows infection or vaccination and presents with multifocal inflammatory lesions, while periodic paralysis causes acute weakness without white matter changes on MRI (Statland et al., 2018; Li et al., 2022). Additionally, reversible splenial lesion syndrome (RESLES/MERS) is often considered by neuroradiologists due to its characteristic transient and isolated lesion in the splenium of the corpus callosum on MRI, which may radiologically overlap; however, it is typically triggered by infection, epilepsy, or metabolic derangement and lacks the associated peripheral neuropathy or family history seen in CMTX1 (Tada et al., 2004; Liu et al., 2017).

In summary, the combination of transient, symmetric white-matter lesions on MRI plus evidence of an underlying peripheral neuropathy (e.g., family history, pes cavus, slowed conduction) is highly suggestive of CMTX1. Early recognition is critical to avoid unnecessary stroke therapies (e.g., thrombolysis) and to prompt genetic counseling.

### 3.4 Atypical presentation and genotype–phenotype

Patients with CMTX1 typically present with peripheral neuropathy due to GJB1 gene mutations, such as progressive muscle atrophy and lower limb weakness. Additionally, some patients exhibit atypical presentations, including delayed motor development, hand tremors, sensorineural hearing loss, pathological fractures, or transient CNS episodes (Yiu et al., 2011; Wang and Yin, 2016). Among these manifestations, CNS episodes are of particular interest. Most reported CNS episodes in CMTX1 are associated with missense GJB1 mutations (often thought to cause gain-of-function). However, our review (Table 1) and others have noted that even frameshift (loss-of-function) mutations can produce identical clinical and MRI phenotypes (Hanemann et al., 2003). For example, both of our cases with frameshift mutations had indistinguishable CNS episodes from those with missense variants. This suggests a poor genotype–phenotype correlation for CNS involvement in CMTX1 (Al-Mateen et al., 2014). These observations challenge earlier studies that specifically linked CNS symptoms to gain-of-function mutations while considering loss-of-function mutations rarely associated (Takashima et al., 2003). The occurrence of reversible CNS episodes across different mutation types suggests a more complex pathophysiology, including genetic modifiers and environmental triggers. The genotype-phenotype correlations uncertainty combined with the atypical clinical presentations significantly complicate the clinical diagnosis of CMTX1. Consequently, clinicians must maintain a high index of suspicion for these non-classical manifestations.

### 3.5 Management and treatment

Currently, there is no disease-modifying treatment for CMTX1, and clinical management remains supportive, focusing on preserving function and slowing disease progression. Core interventions include physical therapy and structured rehabilitation to maintain muscle strength and joint mobility, thereby preventing atrophy and contractures (Lencioni et al., 2015).

Several pharmacologic approaches have been tested in CMTX1. For example, Acceleron’s ACE-083 (a myostatin inhibitor delivered by local muscle injection) increased muscle bulk in CMT patients but failed to improve functional outcomes or quality of life, leading to its discontinuation after Phase 2 (Okamoto and Takashima, 2023). In contrast, gene therapy represents a more promising direction. Preclinical studies using AAV9-based vectors to deliver the GJB1 gene specifically to Schwann cells have demonstrated improved neuropathological and motor outcomes in animal models, though this approach has not yet entered clinical trials (Hertzog and Jacob, 2023).

For the CNS episodes specifically, no acute therapy is established, as they are typically self-limited. Critically, accurate diagnosis is essential to prevent unnecessary and potentially harmful interventions, such as anticoagulants or thrombolytics. Empiric high-dose steroids have been given in case reports (sometimes under the presumption of ADEM) but their efficacy is unclear (Kulkarni et al., 2015; Wu et al., 2015). The key is accurate recognition of CMTX1 to avoid unnecessary stroke interventions. Patients should be counseled on avoidance of triggers: for example, refraining from sudden high-altitude travel or intense hyperventilation when possible, as these may precipitate CNS episodes.

## 4 Conclusion

In conclusion, CMTX1 should be considered in young patients presenting with transient, stroke-like episodes and a distinctive MRI pattern of symmetric, reversible white matter lesions. The presence of clinical signs suggestive of a chronic peripheral neuropathy should further raise suspicion. In such cases, genetic testing for GJB1 mutations is warranted. Once diagnosed, patients should be counseled to avoid known triggers, such as high-altitude travel, to mitigate the risk of recurrent CNS symptoms. However, this study has certain limitations: the number of case reports included was limited, which may affect the statistical power of the findings; there was a lack of standardization in evaluating the potential trigger of “high-altitude exposure”—lack quantitative data such as specific altitude levels, duration, and whether exposure was continuous. Future research should focus on large-scale prospective studies to further elucidate the underlying molecular mechanisms.

## Data Availability

The original contributions presented in the study are included in the article/supplementary material, further inquiries can be directed to the corresponding author.
